# Chemical Changes of Wood Treated with Caffeine

**DOI:** 10.3390/ma14030497

**Published:** 2021-01-21

**Authors:** Patrycja Kwaśniewska-Sip, Magdalena Woźniak, Wojciech Jankowski, Izabela Ratajczak, Grzegorz Cofta

**Affiliations:** 1 Institute of Wood Chemical Technology, Faculty of Forestry and Wood Technology, Poznań University of Life Sciences, Wojska Polskiego 28, 60-637 Poznań, Poland; gcofta@up.poznan.pl; 2 Air Quality Investigation Department, Łukasiewicz Research Network–Wood Technology Institute, Winiarska 1, 60-654 Poznań, Poland; 3 Department of Chemistry, Faculty of Forestry and Wood Technology, Poznań University of Life Sciences, Wojska Polskiego 75, 60-625 Poznań, Poland; magdalena.wozniak@up.poznan.pl (M.W.); izabela.ratajczak@up.poznan.pl (I.R.); 4 Faculty of Chemistry, Adam Mickiewicz University in Poznań, Uniwersytetu Poznańskiego 8, 61-614 Poznań, Poland; wojciech.jankowski89@gmail.com

**Keywords:** caffeine, wood preservative, β-O-4-structure, FTIR, computational study, molecular modeling, DFT

## Abstract

Earlier studies have revealed that wood treated with caffeine was effectively protected against decay fungi and molds. However, there is a need to establish how the caffeine molecule behaves after wood impregnation and how it can protect wood. The objective of the research was to characterize the interaction between caffeine and Scots pine (*Pinus sylvestris* L.) wood as well as to assess the stability of the alkaloid molecule in lignocellulosic material. For this purpose, an elementary analyzer was used to assess the nitrogen concentration in the treated wood. The results showed that caffeine is easily removed from the wood structure through large amounts of water. The changes occurring in the wood structure after impregnation were evaluated with regard to the results obtained by Fourier transform infrared (FTIR) spectroscopy of two model mixtures with caffeine and cellulose or lignin for the purpose of conducting a comparison with the spectrum of impregnated and non-impregnated samples. The observed changes in FTIR spectra involve the intensity of the C=O(6) caffeine carbonyl group and signals from guaiacyl units. It might indicate favorable interactions between caffeine and lignin. Additionally, molecular simulation of the caffeine’s interaction with the guaiacyl β-O-4 lignin model compound characteristic for the lignin structure using computational studies was performed. Consequently, all analyses confirmed that caffeine may interact with the methylene group derived from the aromatic rings of the guaiacyl group of lignin. In summary, scanning electron microscope (SEM) observations suggest that caffeine was accumulated in the lignin-rich areas of the primary walls.

## 1. Introduction

As a natural biopolymer, wood is characterized by many unique features including its physical properties and structure. Wood has the ability of resilient and plastic deformation under the influence of external mechanical forces. In practice, material such as trunks, branches, and roots are broadly used in the construction, furniture, paper, and bioenergetics industries [1,2,3,4].

The main constituents of wood are cellulose, hemicelluloses, lignin, and other organic compounds like waxes, fats, and resins [5]. Wood is a natural chain polymer that contains about 40–45% cellulose with different lengths of cellulose chains, depending on various wood species. It is composed of D-glucopyranose residues that are bound by β-(1-4)-glucosidic bonds [6,7,8,9]. Besides cellulose, the most prevalent polymeric organic substance in wood structure is lignin (approx. 20 to 40%) [6,7]. The complex structure of lignin consists of an irregular array of variously bonded hydroxyl- and methoxy-substituted phenylpropane units. The primary components of wood structure also include polysaccharides with a low degree of polymerization and more branched chains called hemicelluloses [10]. In light of its numerous applications, also in external conditions, wood is exposed to various microorganisms and varying atmospheric conditions. The polysaccharides are mainly responsible for moisture uptake and release in changing atmospheric conditions and consequently for biological degradation [11,12].

Most of all, the primary problem with using wood is its susceptibility to the destructive action of microorganisms. Wood deterioration is caused by three types of microorganisms that inhabit the cells of wood by metabolizing its components: wood-destroying fungi; wood-staining fungi, and molds and bacteria [13]. The protection of wood against fungi is mainly based on chemical preparations. The use of long-standing biocides such as wood preservatives is rarely compatible with environmental protection strategies. As has been established, biocides and other wood-preserving chemicals have become long-term environmental pollutants due to their chemical stability [13]. Modern wood preservatives are required to effectively target specific groups of organisms, show no adverse effects on humans, animals, and the natural environment, and not accumulate in organisms. Of significance is the development of eco-friendly biocides with a low impact on the natural environment as well as progress in the modification of wood toward a permanent immobilization of these chemicals in the wood structure to prevent them from leaching into the environment [14,15,16]. The use of natural bioactive compounds is an attractive approach in wood preservatives [17]. That said, the replacement of traditional and toxic substances requires time to develop, and the methodology must be based on knowledge about such substances’ properties, mechanism of action, and toxicity for humans and the environment.

An important aspect in the development of new agents in wood protection is knowledge about the mechanism of binding with the wood material. Wood can be analyzed by a combination of instrumental techniques commonly used in polymer science. However, the chemical composition of wood as well as its highly anisotropic nature do not facilitate the characterization of chemical changes. FTIR spectrometers have been used frequently to analyze the chemical changes taking place in wood after a fungal attack or environmental exposure [6,18,19]. The broad application of FTIR in the quantitative and qualitative analysis of wood is due to its ability to provide information about the presence of functional groups, composition, and some specific structural features.

The search for new and environmentally friendly solutions used to extend the natural durability of wood still remains a very current issue due to legal restrictions and requirements for new chemical wood preservatives. This paper attempts to determine the effectiveness of caffeine binding to wood material. Earlier results have proven that after impregnation with caffeine, wood was effectively protected against decay fungi and molds [20,21]. However, the resistance of the treated wood subjected to leaching was lower. Understanding the changes occurring in the structure of the protected wood has allowed us to evaluate the stability of the bond between the alkaloid and the lignocellulosic material. This could help to extend the use of caffeine for the protection of wood products for outdoor application.

The core objective of the research was to characterize the interaction between caffeine and pine wood (*Pinus sylvestris* L.) as well as to assess the stability of the alkaloid molecule in lignocellulosic material. In order to determine the structural changes in wood treated with caffeine subjected to natural and accelerated aging, Fourier transform infrared (FTIR) spectroscopy was applied. Scanning electron microscope (SEM) analysis was used to establish where the caffeine molecules are located in the wood structure. An elemental analyzer was applied to estimate the degree of the leaching of nitrogen, which is a part of the alkaloid molecule from the treated wood. Additionally, the bonding between the caffeine and the β-O-4 model structure was simulated using computational studies.

## 2. Materials and Methods

### 2.1. Wood Specimens

Test specimens (40 ± 0.5) × (40 ± 0.5) × (5 ± 0.5) mm^3^ (longitudinal × radial × tangential) with nine to thirteen annual growth rings per 10 mm and an average density of 540 kg m^−3^ (average moisture content was 8%) were prepared from Scots pine (*Pinus sylvestris* L.) sapwood originating from the Wielkopolska Province. The samples were without knots and free of visible evidence of resins; they also showed no biotic infection. Scots pine is a basic species in Polish forests and is usually used in outdoor construction. The sapwood of pine is easy to process, is soft, and easily absorbed. As a result, it has become a very popular and widely used raw material. In order to obtain the initial mass before impregnation, the samples were oven-dried (24 h, 103 °C).

### 2.2. Chemicals

Caffeine (CAS no. 58-08-2) in the form of white powder was purchased from Sigma−Aldrich, St. Louis, MO, USA. As instrumental research methods were applied, we used caffeine of high purity. Further study involved the use of cellulose fibers (medium, CAS no. 9004-34-6) and Kraft lignin powder (CAS no. 8068-05-1). Both substances were purchased from Sigma-Aldrich, St. Louis, MO, USA.

### 2.3. Preparations of Wood Specimens

The samples were impregnated in a caffeine solution of 20 mg/mL for 15 min under a vacuum of 0.08 MPa, followed by soaking for 2 h under atmospheric pressure at room temperature [14]. Deionized water was used to prepare working caffeine solutions. The solutions were homogenous. The retention of caffeine was 14.7 kg/m^3^.

Part of the samples was subjected to leaching for outdoor applications according to EN-84 [22]. The treated wood samples were impregnated with deionized water under vacuum conditions of 0.08 MPa and soaked in deionized water for two weeks. During this time, the water was changed 10 times.

### 2.4. Chemical Analysis

#### 2.4.1. Elementary Analysis

The treated samples were dried to a constant weight at 103 ± 2 °C. The same was done with samples after leaching. Both samples in the form of sawdust were weighed (2–3 mg) in zinc vessels. The content of nitrogen, carbon, hydrogen, and sulfur was determined using the FLASH 2000 Series analyzer (Thermo Fisher Scientific, Waltham, MA, USA). The instrument was calibrated with BBOT (2,5-bis-(5-tert-butyl-benzoxa-zol-2-l)thiophene), (Thermo Fisher Scientific, Waltham, MA, USA) using linear matching. A five-point calibration curve was prepared for each element. The correctness of curves was verified by certified reference materials *Alfalfa* and *Birch leaf* (Elemental Microanalysis Ltd., Okehampton, UK). Elemental analysis was used to determine the degree of leaching of nitrogen as a characteristic element of the caffeine molecule. The parameters of the elemental analyzer are shown in Table 1.

#### 2.4.2. Fourier Transform Infrared (FTIR) Analysis

Powder samples were mixed with KBr (Sigma-Aldrich, St. Louis, MO, USA) at a 1/200 mg ratio. Spectra were registered using the Infinity spectrophotometer (Mattson Technology, Fremont, CA, USA) with a Fourier transform at a range of 600–4000 cm^−1^ at a resolution of 2 cm^−1^, registering 64 scans. The FTIR analyses were conducted for basic spectra. Band intensity was determined from the measured absorbance in the peak maximum, taking into consideration the base line plotted separately for each peak.

For chemical analysis, pine control samples, impregnated samples, and non-impregnated samples after natural and accelerated aging were ground using a laboratory mill (Ika Works Inc., Staufen im Breisgau, Germany). Wood was used in the form of a dust fraction of less than 0.2 mm in diameter. Additionally, a pure caffeine spectrum was performed to identify significant bands.

The reference material was prepared for a better interpretation of the effects occurring in wood impregnated with caffeine. To compare the FTIR spectra of treated wood, two reactions of the main wood components (cellulose and lignin) with caffeine were prepared. The reaction mixture consisted of caffeine (20 mg/mL) in distilled water and either cellulose fibers or lignin (50 mg/mL). Kraft lignin was chosen because of its pH equal to 6.5 to avoid affecting the reaction trend. All reagents were mixed for 24 h (500 rpm, 23 °C) and centrifuged for 20 min (2500 rpm, 23 °C). Subsequently, the centrifuged liquid and a solid mixture were separated through decantation. The obtained reaction products were conditioned on a watch glass in a desiccator with silica gel up to constant mass.

#### 2.4.3. Scanning Electron Microscopy of the Treated Sample

The studies of wood structure after treatment were carried out by means of a scanning electron microscope (SEM) JEOL 7001F (SEI detector, maximum 30 kV accelerating voltage, Tokyo, Japan). Before the experiments, samples were well dried and sputtered with a thin layer of gold.

### 2.5. Computational Studies

This study calculated the interactions between the caffeine molecule and models for lignin (αR, βR guiacyl β-O-4 structure). This method has been shown to accurately predict a broad range of noncovalent interactions at modest computational expense.

Molecular Dynamics simulations consisted of caffeine and αR, βR guiacyl β-O-4 structure (as proposed by Besombes et al. (2004) as the lignin model), and were performed assuming the CHARMM36 force field, which is often used for such simulations [23,24]. Each molecule was parametrized with the use of the PARATOOL [25] software (i.e., a plug-in for the molecular viewer VMD (Visual Molecular Dynamics) and distributed with VMD [26]. The molecular geometry of each compound was optimized using the B3LYP/6-31+G(d) level of theory [27]. Natural population analysis (NPA) and frequency analyses [28] were used to obtain a set of point charges and a Cartesian Hessian matrix. The calculations were performed with the Gaussian 09 software [29]. On the basis of these calculations and the use of PARATOOL, the parameters for the investigated molecules were obtained. Molecular Dynamics simulations were performed in the TIP3P (Transferable Intermolecular Potential with 3 Points) water box [30]. Electrostatic and Van der Waals interactions were treated with a cutoff of 12 Å. The first step of each simulation was minimization of the structure’s energy, which was carried out in 1 ns. Energy minimization was followed by simulated annealing for 1.4 ns with a temperature rising from 250 K to 600 K and then decreasing back to 250 K. The last step of the simulation was the production run during which the temperature was kept at 298 K by applying the Langevin [31] forces to all heavy atoms with a damping coefficient of 1 ps^−1^. The production run lasted 60 ns. All simulations were performed in Nanoscale Molecular Dynamics NAMD 2.99. To assess the stability of the investigated system, we calculated RMSD (root- mean-square deviation). For the investigated compounds, the calculated RMSD was used to group conformers into five clusters, with the use of the clustering plugin of VMD, which uses the Quality Threshold algorithm [32]. To assess the interaction energy of each complex, a similar methodology as presented by Trujillo et al. (2016) [33] was used. After completing simulations and grouping conformers to five clusters, the interaction energy between compounds in a vacuum and an aqueous solution was calculated (the average structure calculated on the basis of the simulation trajectory). Energy calculations were carried out with the WB97XD method and in the 6-31++G(d,p) basis set, which are recommended for non-covalent interactions by Chai et al. (2008) [34]. To take into account the effect of the aqueous solvent, we used the Polarizable Continuum Model (PCM).

### 2.6. Statistical Analysis

A statistical analysis was conducted using the Statistica 10.0 software with the analysis of variance (ANOVA) applying Tukey’s honest significant difference test (Tukey’s HSD). One-way comparison tests were performed at a 0.05 significance level.

## 3. Results and Discussion

### 3.1. Elementary Analysis 

In the first stage of the research process, the percentage content of nitrogen according to elementary analysis (coming from amine groups present in the caffeine) indicates the presence of caffeine in the sample. Table 2 shows the nitrogen, carbon, and hydrogen content in wood after impregnation with caffeine as well as the content of these elements in the treated wood after leaching. As it can be seen, changes in the content of individual elements were significant only in the case of nitrogen. The nitrogen content for the impregnated sample was 0.98%, while for the untreated sample it was 0.07%. 

The nitrogen content in the wood before and after leaching was 0.98% and 0.47%, respectively. These results showed a significant difference of caffeine content in unleached and leached wood. The degree of caffeine leaching from treated wood was 52.14%. Consequently, it can be concluded that caffeine is easily removed from the wood structure through large amounts of water. Moreover, referring to previous research results in Kwaśniewska et al. (2018), the retention of caffeine was about 14.7 kg/m^3^ [20]. It was statistically proven that the samples with a retention of caffeine at approximately 7 kg/m^3^ showed resistance to decay after the leaching procedure. This retention corresponds to half the concentration of the impregnation solution and the results of the elemental analysis. The obtained results suggest that caffeine should be used in wood preservative products for applications with no direct contact to rain, where leaching is reduced. The risk of wood becoming wet in typical external applications makes caffeine unsuitable for wood preservation. Our studies so far indicate that this proposed protection could be dedicated only to the first and second use classes of wood concerning all internal construction timber within the building envelope.

### 3.2. FTIR Analysis

#### 3.2.1. Analysis of Pure Caffeine

**Figure 1:**
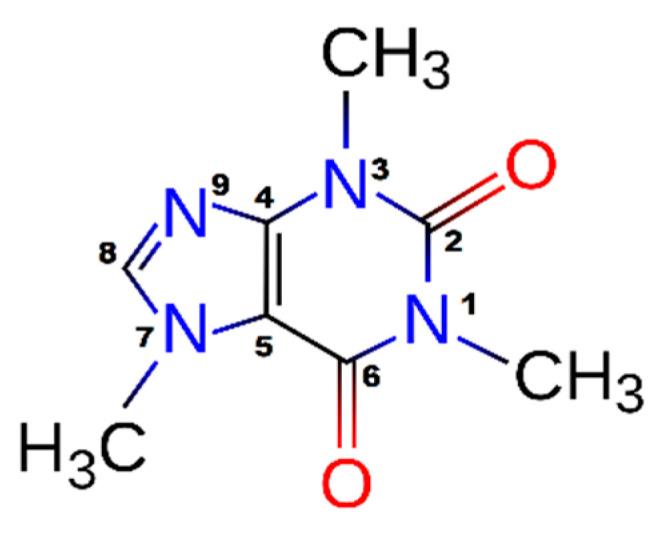
The molecular structure and atomic labeling for caffeine.

The caffeine molecule presented in Figure 1 comprises ten C−H bonds (including nine C−H bonds from methyl groups), ten C−N bonds, two C=O carbonyl groups in the ring position, one C=C bond, one C=N bond, and one C−C bond [35]. As with other purines, caffeine is characterized by a planar surface surrounded by hydrophobic and hydrophilic functionalities. This is because of two groups: the hydrogen C8−H8 donor group capable of forming hydrogen bonding makes the molecule slightly soluble in water, while hydrogen bonding acceptor groups O2, O6, and N9 form the hydrophilic groups [36].

**Figure A1:**
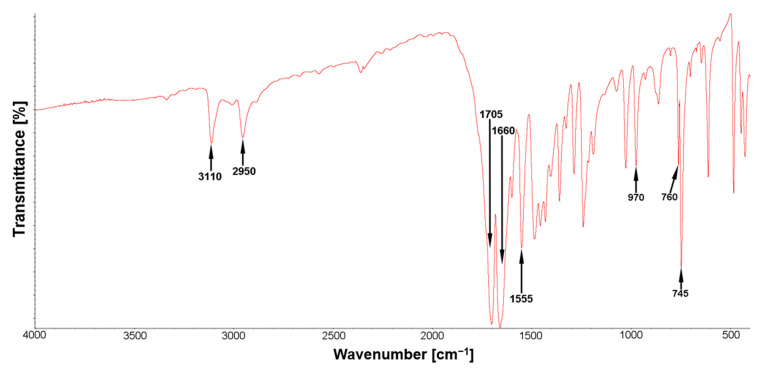
Fourier transform infrared (FTIR) spectra of pure caffeine at the 4000 to 400 cm^−1^ range.

The spectra of pure caffeine with a spectral range from 4000 cm^−1^ to 400 cm^−1^ and the corresponding bands assignments are provided in Figure A1 (see Appendix A). Before conducting a comparison, we summarized the majority of signals in the caffeine spectrum. The caffeine FTIR spectrum showed the characteristic two peaks of the vibrations of the C–H (2950–2850 cm^−1^) methyl groups [37]. This asymmetric vibration enables the quantitative analysis of caffeine in extractives of coffee beans or tea leaves [38]. Interesting features concern the caffeine bands at 1705 cm^−1^ and 1660 cm^−1^ assigned to the stretching vibration energy of conjugated C=O(2) and C=O(6) carbonyl groups [35]. In the spectrum range from 1600 cm^−1^ to 1400 cm^−1^, there are vibrations of C=N and C=C bonds, while in the spectrum range from 1330 cm^−1^ to 1000 cm^−1^, there were bending vibrations in the plane from the C−H, C−N, and C−C bonds. A medium peak observed at 1350 cm^−1^ may be attributed to the stretching vibration of the C−N bonds of caffeine. In addition, peaks at 1200 cm^−1^ also fell in the spectral region of the stretching vibration of the >C=O ketonic carbonyl group and C−N bonds. A minor peak observed at 860 cm^–1^ and 1025 cm^–1^ may be due to the stretching of the C−C bond of caffeine [39].

#### 3.2.2. Analysis of Cellulose after Reaction with Caffeine 

**Figure 2:**
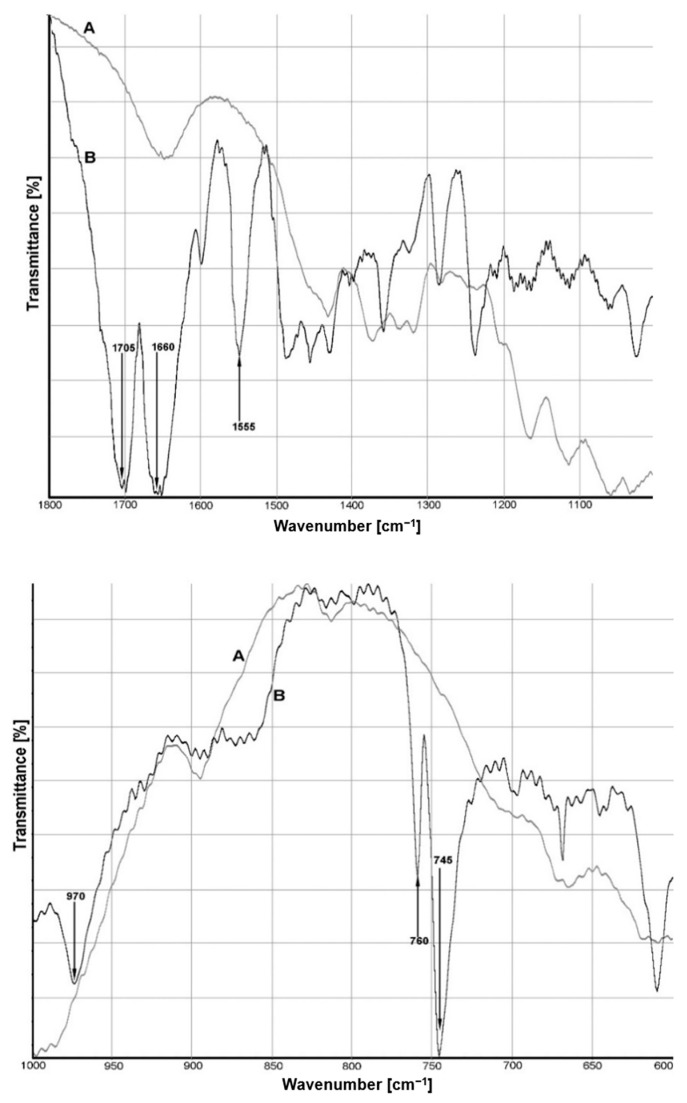
Fourier transform infrared (FTIR) spectra of cellulose fiber (A) and the mixture of cellulose with caffeine (B) at the 1800 to 1000 cm^−1^ range, and at the 1000 to 500 cm^−1^ range.

**Figure A2:**
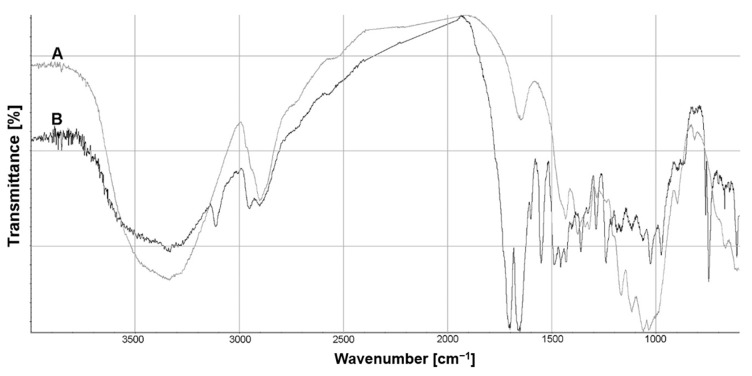
FTIR spectra of the mixture of cellulose (A) and the mixture of cellulose with caffeine (B) from the 4000 to 400 cm^−1^ range.

The FTIR spectra of the mixture of cellulose with caffeine at the 1800–1000 cm^−1^ and the 1000–500 cm^−1^ ranges are presented at Figure 2. The holistic infrared spectrum of the mixture with caffeine and cellulose fibers (see Appendix A, Figure A2) showed peaks typical of a cellulosic substrate. At around 3400 cm^−1^, it can be observed that there was a strong broad band corresponding to different O–H stretching modes [40]. 

The spectrum showed that the band in this frequency decreased when cellulose interacted with caffeine. The caffeine structure contains several lone pairs of electron donation sites. This offers the ability to interact with the host by donor–acceptor interactions in addition to the specific binding through hydrogen bonds [41]. Earlier studies have shown that the dominant site of protonation is expected to be at the nitrogen N(9) position. Usually, the water molecule is bonded to the nitrogen atom of the imidazole ring of caffeine. However, the reduced intensity of the band at 3400^−1^ cm may indicate a partial loss of intramolecular hydrogen bonds in cellulose. 

The two peaks between 2950 cm^−1^ and 2850 cm^−1^ are associated with the asymmetric and symmetric C−H methyl and methylene stretching groups present in the spectra for cellulose in Figure A2. The bands at 1665–1645 cm^−1^ in the cellulose spectrum have been reported to be a hydrogen bonding coupled with COO¯ of the cellulose [40]. The vibrations at around 1600 cm^−1^ and 1555 cm^−1^ have a high pyramidic character in caffeine and decreases the stretching vibrations (ʋ) of the C=C and C=N bond. The vibrations of the ʋ(C=N) band in the imidazole structure are also observed at 1490 cm^−1^. The spectrum region from 1455 cm^−1^ to 1405 cm^−1^ represents the C−H and C=C bond vibrations derived from the caffeine molecule. The band at 1375 cm^−1^ originates from the deformations of the cellulose CH_2_ group. Interesting changes were observed when the spectrum of the prepared mixture was compared with the spectrum of caffeine. The intensity of the band occurring at 1285 cm^−1^ decreased. Additionally, this band showed a small downward shift in wavenumber in the IR spectrum for the mixture of caffeine with cellulose (1282 cm^−1^). This band may show a movement of C−H bending vibrations in cellulose or vibrations in the ʋ (CH_3_) bond plane in the caffeine ring [42]. In the fingerprint region, the bands at 970 cm^−1^ correspond to the rocking vibrations of the CH_3_ group in the caffeine molecule. In addition, the bands at 760 cm^−1^ and 745 cm^−1^ are represented by bending vibrations outside the plane of the carbonyl group C=O.

In the present study, we did not succeed in identifying the corresponding band of caffeine with cellulose fiber that would prove the permanent binding of both substances. Moreover, we observed that the slight shift of the band in the region of 1285 cm^−1^ is probably due to various interactions of caffeine’s internal vibration ʋ (CH_3_). Referring to the work of Taeye et al. (1985), regarding hydrogen bonding via proton donors, we know that when hydrogen bonds occur at an exocyclic atom of a polyfunctional base, the in-plane and out-of-plane ring modes are not shifted by more than 2 cm^−1^ [43]. However, hydrogen bond complexes between nitrogen or oxygen were not observed.

#### 3.2.3. Analysis of Lignin after Reaction with Caffeine

**Figure 3:**
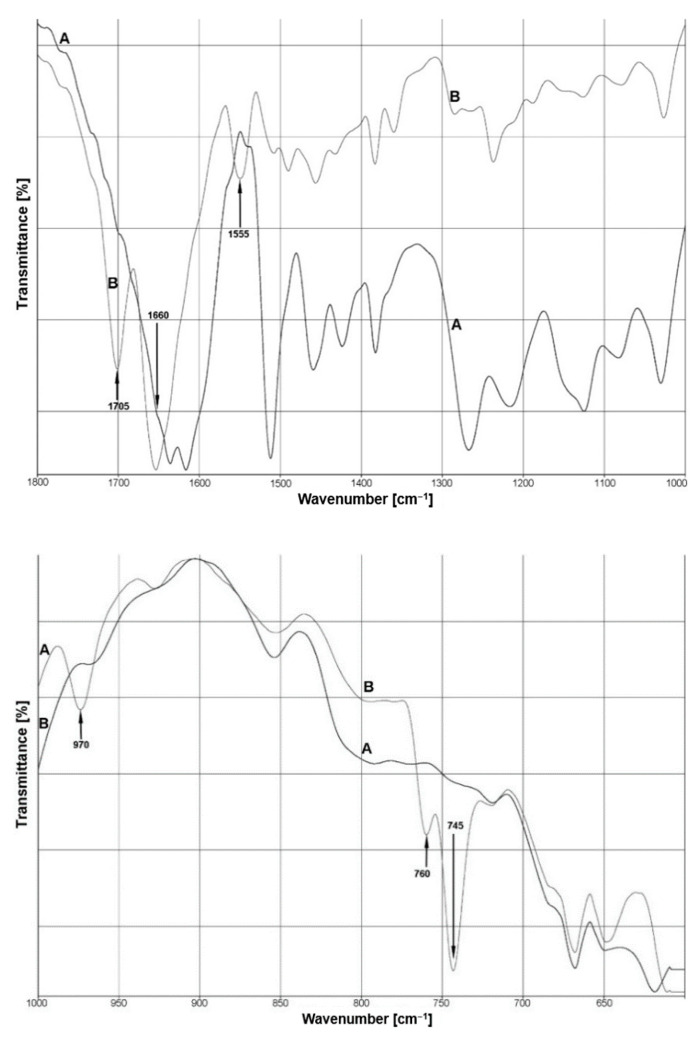
FTIR spectra of lignin (A) and the mixture of lignin with caffeine (B) at the 1800 to 1000 cm^−1^ range and at the 1000 to 500 cm^−1^ range.

The changes in the intensity of the bands and their frequency in the infrared spectra of lignin and the lignin–caffeine mixture are shown in Figure 3. Depending on the origin and the applied isolation process, lignin contains different functional chemical groups in various amounts. We can indicate such groups as hydroxyl (phenolic or alcoholic), methoxy, carbonyl, and carboxyl groups [44]. Kraft lignin is obviously different from native lignin in chemical composition and structure. It is characterized by lower molecular weight through the cleavage of α-O-4 and β-O-4 linkages [45]. 

The spectrum (Figure 3) is shown as a sum of all individual vibrations with the appearance of several new modes or noticeable peak shifts, indicating the presence of interactions. The bands at 1705 cm^−1^ and 1660 cm^−1^ can be assigned to the energy of stretching vibrations of the caffeine carbonyl groups, respectively, C=O(2) and C=O(6). The intensity of the first ʋ(C=O) band decreased for the lignin–caffeine spectrum. Lignin-associated high bands were visible at 1600 cm^−1^, 1514 cm^−1^, 1460 cm^−1^, and 1269 cm^−1^ for pine [46]. The bands at 1600 cm^−1^ and 1514 cm^−1^ were assigned to aromatic skeletal vibration bands. The intensity of vibration at 1514 cm^−1^ was significantly decreased. 

Subsequent bands of reduced intensity at 1460 cm^−1^, 1425 cm^−1^, 1380 cm^−1^, 1280 cm^−1^, and 1214 cm^−1^ represent different bands and peak ratios derived successively from vibrations of C=C, C−O, C−H, and C=O bonds characteristic for lignin [47], while the C–H deformations of the methyl and methylene groups combined with aromatic ring vibration were at a position of 1460 cm^−1^. The region below 1300 cm^−1^ was more difficult to analyze but the typical bands could still be examined. Bang vibrations assigned to guaiacyl units in the lignin decreased, specifically the bands at 1280 cm^−1^ and 1214 cm^−1^. Additionally, the band from C−O stretch vibrations at 1150 cm^−1^ associated with the deformations of aromatic C−H in the aroma plane were derived from the guaiacil ring [45]. Moreover, the IR spectrum region between 1300−1200 cm^−1^ had a pronounced downward shift in the wave number of the lignin structure interacted with caffeine. This described effect and the changes in the intensity of the C=O(6) caffeine carbonyl group may signify a weakening of the self-assembling of caffeine and evidence advantageous interactions with lignin. At the 1000 cm^−1^ to 500 cm^−1^ range in the spectrum, there were visible bands derived from the characteristic vibrations of caffeine (970 cm^−1^, 760 cm^−1^, and 745 cm^−1^).

#### 3.2.4. Analysis of Treated Wood

**Figure 4:**
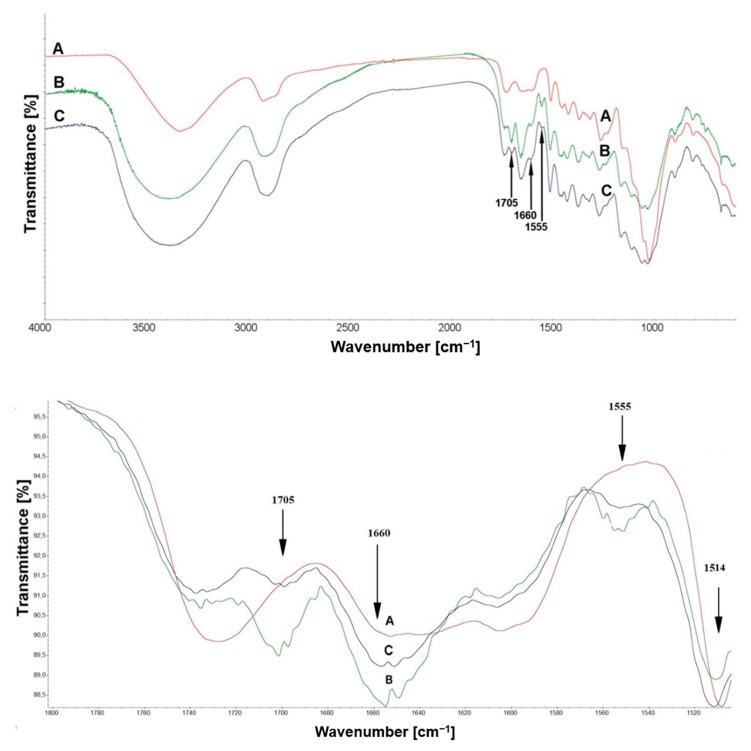
FTIR spectra of Scots pine (A) and wood treated with caffeine (B), and treated wood after leaching (C).

In order to compare the spectral changes caused by the effects of caffeine on Scots pine, Figure 4 presents the spectra of wood treated with a caffeine solution before and after leaching.

The spectrum of the treated wood showed characteristic bands assigned to the caffeine molecule at 1705 cm^−1^, 1660 cm^−1^, and 1555 cm^−1^. The intramolecular hydrogen bond was observed at around 3400−3300 cm^−1^. This is also related with the OH association of cellulose, functional groups, and the aromatic system of lignin. Furthermore, in the spectrum region, the bands at 1600 cm^−1^, 1510 cm^−1^ and 1120 cm^−1^ were assigned to C=O, C–H, and C–O deformation, bending, or stretching vibrations of different groups in the lignin hydrocarbon chain [18]. The C−O−C band at 1160 cm^−1^ is known to be sensitive to changes in cellulose crystallinity; after impregnation, the band remained unchanged [48]. The FTIR spectrum (Figure 4), which showed no obvious appearance of new band vibrations in these positions as well as no shifts in peaks, demonstrated the lack of a permanent bond between caffeine and chemical components of wood. However, after comparing the changes in wood material to the lignin spectrum after caffeine impregnation, we found some similarities. The bands with a reduction in intensity in previous analysis were visible in the spectrum below 1514 cm^−1^ or 1280 cm^−1^ and 1214 cm^−1^. These band vibrations were assigned to aromatic skeletal vibration and guaiacyl units in the lignin. The intensity of the band at 1514 cm^−1^ after impregnation of Scots pine was particularly visible, but this change did not persist after leaching. FTIR analysis has emerged as a basic and versatile analytical technique to study not only plant development, but also the cell wall composition and organization. It is also an important method to investigate the chemical changes in wood structure after further processing [49,50]. The usefulness of using FTIR in wood chemistry stems from its ability to provide information about the presence of functional groups, composition, and some specific structural features. Furthermore, in combination with other research methods such as computational studies and SEM analysis, it can provide plenty of information on the possible interactions between a caffeine molecule and wood material

Additionally, in the case of samples treated with caffeine after the leaching procedure, there were noticeable changes in the intensity of the bands characteristic of caffeine vibrations at 1705 cm^−1^, 1660 cm^−1^, and 1555 cm^−1^. The intensity of these bands decreased for impregnated samples after leaching. This is related to caffeine leaching from the wood structure.

A quantitative analysis with changes in the absorption intensity of the bands at 1705 cm^−1^, 1660 cm^−1^, and 1555 cm^−1^ was conducted. All the intensities of peaks were normalized to the intensity of the 1514 cm^−1^ band, which was selected as stable in the spectra. Even if the chosen band did not remain completely constant in the previously described spectra due to skeletal aromatic rings stretching in lignin, it represents one of the less variable bands during wood impregnation and aging. All data are summarized in Table 3. A reduction of intensity was observed for all bands. The significant changes confirmed by comparable relative absorbance values within the N−H group may indicate a leaching effect of the caffeine.

### 3.3. Computational Studies

Molecular modeling at the supramolecular level is an excellent tool for providing valuable conformational information in the study of lignin. There are not many computational analyses that have targeted lignin or lignocellulosic structures and their capability of interactions [41,51,52]. Considering the complexity of the lignin network and the ability to form bonds with fungicides, we decided to calculate the interactions between the caffeine molecule and the structural part of lignin (αR, βR guiacyl β-O-4 structure). Indeed, for the purpose of studying the conformational features of a lignin polymer, the β-O-4 junction structure is an excellent model system that represents the dominant bonds between units in lignin [23,53]. Simulations allowed us to find the potential energy minima corresponding to the studied system and clustering analysis helped identify five clusters based on the simulation trajectory. In all the obtained clusters, in only one case (cluster 1) was the caffeine and the β-O-4 structure close enough to observe their interaction. The coordinates of all the obtained structures are included in Table A1, Table A2 and Table A3 (see Appendix B), but in a further part of the discussion of results, only one structure with a visible interaction will be presented.

**Figure 5:**
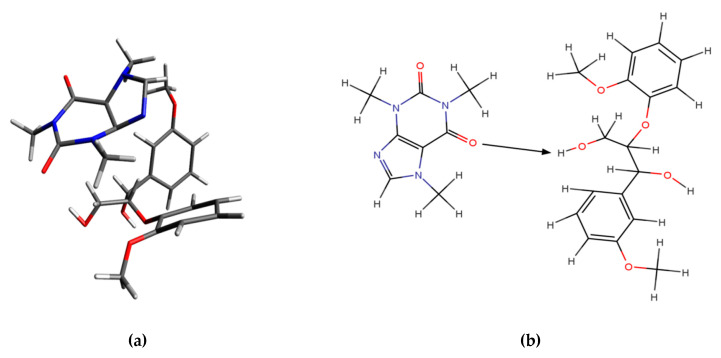
Structure (**a**) and scheme of the interaction (**b**) of cluster 1 in a system consisting of αR, the βR guiacyl β-O-4 model compound, and caffeine.

This structure was studied at the QM level to find interaction energy. The results in a vacuum and an aqueous solvent are summarized in Table 4 (an extended version of Table 4 is included in Table A3). According to DFT calculations for cluster 1, the energy interaction between αR, the βR guiacyl β-O-4 structure, and caffeine was −11.0 kcal/mol in vacuo and −11.4 kcal/mol in a water solvent. The structure of cluster 1 is depicted in Figure 5a and the scheme of interaction in this system is shown in Figure 5b.

Geometries from DFT calculations in which the caffeine molecule approaches the structural part of lignin by simulating the bond trajectory between the methylene group and caffeine carbonyl group C=O(6) were optimized. In cluster 1, the structure of caffeine and the αR, βR guiacyl β-O-4 model compound caffeine could interact with the hydrogen atom of the methylene group via the O6 oxygen atom. The distances between the oxygen atom and the hydrogen atom was 2.185 Å. In addition, the calculations in the aqueous solution showed that the extent of molecular interactions was considerably reduced in comparison to simulations in a vacuum.

In the literature, there are interesting reports on the applications of cellulose and caffeine in the biomedical and cosmetic areas. Due to the properties of caffeine such as the ability to swell, biocompatibility, and nontoxicity, polysaccharides including cellulose have been often used to encapsulate caffeine [54]. Caffeine is a broadly studied drug that contributes to the fast release and weak interactions of bio-cellulose nanofibrils [39]. Hardy et al. (2007) described caffeine as a simulator of hydrophilic drug release systems because it easily diffuses out of the polymer tablet without a chemical interaction with the cellulosic substrate [55]. Hydrogen bonding or other chemical interactions between caffeine and cellulose also do not occur elsewhere in the research area. The results obtained by Lavoine et al. (2014) show that caffeine was completely released from the paper additionally coated with microfibrillated cellulose [56]. However, in other investigations, the authors concluded that caffeine was trapped in the entanglement of nanofibers and this effect depends on the molecular weight and steric hindrance of active molecule [57]. These initial results create evident further research opportunities for applications of molecules with antimicrobial properties in the food packaging field. As chemical preservatives are not recommended in contact with food in light of the current demand for alternative choices, natural substances are being considered.

**Figure 6:**
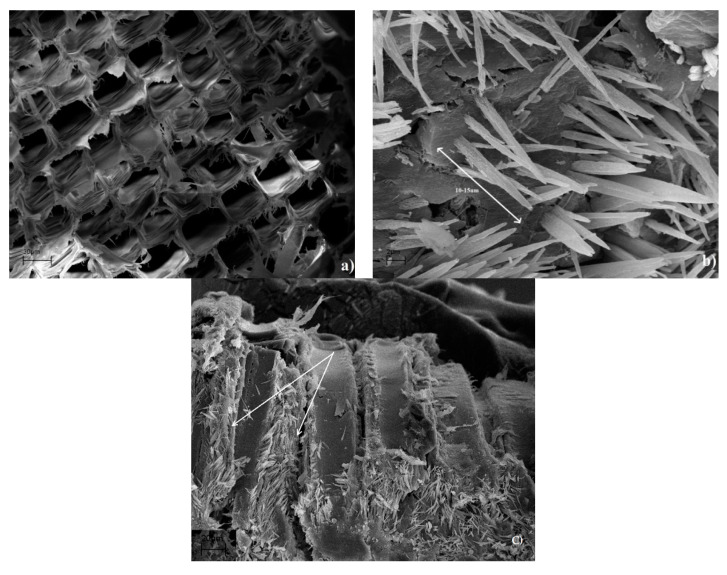
Scanning electron micrographs of wood treated with caffeine (**a**) cross-section, (**b**) needle-shaped caffeine crystals, (**c**) radial cross-section, cellulose fibrils.

On the other hand, the coffee bean also has a lignocellulosic structure. The chemical composition of the waste coffee pulp contains, among others, 33.85% cellulose, 1.24% hemicellulose, 9.40% lignin, and 0.81% caffeine, etc. [58]. The presence of this alkaloid in beans and young leaves explains the possible role of caffeine as a chemical defense. Breda et al. (2013) visually determined that caffeine was on the periphery of cells of xylem and phloem. They concluded that it might be possible that caffeine is bound with the cell wall phenolic compounds. During immunolocalization, caffeine washed out easily and the tissues that were poor in phenolic cell wall components produced a weaker signal due to a lower caffeine content [59]. A similar phenomenon to that described above was observed in the case of wood impregnation. The middle lamella and primary wall are mainly composed of lignin (84%) [8]. These SEM images (Figure 6a) showed ineffective penetration of the cell wall by caffeine, since there were no traces of caffeine in the cell lumen. Only the formation of aggregates or crystallized caffeine was observed. Caffeine has the ability to dissolve in high dielectric solvents with a high dielectric constant, but upon drying, the caffeine tend to crystalize. The same phenomenon is visible in Figure 6b. SEM images show needle-shaped crystals (10–15 µm) of caffeine grown on cell walls after the evaporation of distilled water. As described by Furtado et al. (2020), the crystallization effect is related to the self-organization of caffeine molecules through the π-stacking interaction effect [54]. The SEM observations suggest that caffeine tends to fill intercellular spaces. Caffeine was accumulated between cellulose fibrils visible in the radial cross-section (Figure 6c). On the other hand, it is interesting that the calculated molecular size of caffeine is 0.78 nm in length. As is known, the secondary cell wall alone has a lateral thickness of 2 to 3 nm [60]. The exact morphology is not yet fully explained, but we can initially conclude that the penetration of caffeine into the cell wall cannot progress through spherical blockades.

## 4. Conclusions

1. Caffeine is significantly leached from the wood structure after aging. This was evidenced by the reduced (about 52%) content of nitrogen in the treated wood subjected to leaching in comparison to the unleached treated wood. A low penetration of caffeine led to an accumulation of caffeine in the wood surface, which also leached out more easily.
2. The FTIR analysis demonstrated that there were no chemical interactions between caffeine and cellulose. No relevant changes and no visible bands from new vibrations resulting from the interaction of caffeine with cellulose were observed.
3. The changes observed in the FTIR spectra involved the intensity of the C=O(6) caffeine carbonyl group and the signals from guaiacyl units. This might indicate a weakened self-assembling of caffeine molecules and evidence favorable interactions between caffeine and lignin. It might be possible that caffeine is bound or in complex with the cell wall phenolics. Various vibrations common for the lignin structure changed after treatment with caffeine.
4. The results of molecular modeling demonstrate that these interactions occurred between the carbonyl (C=O) groups of caffeine and the methylene group on the guaiacyl aromatic ring. The results of theoretical research on the interactions of caffeine with the guaiacyl β-O-4 model compound showed that the distance between oxygen (O6) and hydrogen was 2.185 Å. Additionally, the SEM observations suggest that caffeine was accumulated in the middle lamella and the primary walls, which were composed mainly of lignin.

## Figures and Tables

**Table 1:** Parameters of the elemental analyzer.

Parameter	CHNS Analysis
Oven temperature	950 °C
Chromatographic column temperature	65 °C
Gas flow	He–140 mL min^−1^ O_2_–250 mL min^−1^
Time of analysis	660 s

**Table 2:** Content of elements in treated wood and in wood after leaching.

Samples	Content of Element [%]		
	N	C	H
Untreated	0.074 ^a^* ± 0.006	47.389 ^a^ ± 0.081	6.309 ^a^ ± 0.027
Unleached	0.980 ^c^ ± 0.041	47.592 ^a^ ± 0.006	6.259 ^a^ ± 0.042
Leached	0.469 ^b^ ± 0.003	47.536 ^a^ ± 0.070	6.309 ^a^ ± 0.174

S < 0.01, * values in the same column followed by the same letter (^a^, ^b^, ^c^) were not significantly different by Tukey’s honest significant difference test (Tukey’s HSD) (*p* < 0.05).

**Table 3:** Average value of relative absorbance of wood samples. All values were normalized to 1514 cm^−1^.

Samples	Band Frequency (cm^−1^)		
	A_1705/1515_ ν C=O	A_1660/1515_ ν N−H	A_1555/1515_ ν C=N
Control	-	-	-
Unleached	0.9290 ^b^*	1.0919 ^a^	0.8082 ^c^
Leached	0.9153 ^b^	0.9040 ^b^	0.7768 ^c^

* Values in the same column followed by the same letter do not differ significantly according to Tukey’s honest significant difference test (Tukey’s HSD) (*p* < 0.05).

**Table 4:** Interaction energy between αR, the βR guiacyl β-O-4 model compound, and caffeine in the obtained average cluster structure.

	Interaction Energy [kcal/mol]	
System Consisting of αR, the βR Guiacyl β-O-4 Model Compound and Caffeine	In Vacuo	In an Aqueous Solution
Cluster 1	−11.0	−11.4

## Data Availability

The data presented in this study are available on request from the corresponding author.

## Appendix B

**Table A1:** Atomic coordinates of the system consisting of αR, βR guiacyl β-O-4, and caffeine: cluster 1 and cluster 2 structures.

Atomic Coordinates of Gu_Caf Complexes						
	Cluster 1			Cluster 2		
Atom	x	y	z	x	y	z
C	−15.05000	−7.18400	−13.37900	−9.45900	−2.00800	−3.12100
C	−15.53900	−6.87100	−14.70700	−8.48800	−2.15400	−4.15500
C	−13.95200	−6.47400	−12.77300	−9.99700	−3.28400	−2.54300
C	−13.61900	−5.10600	−13.27700	−9.09500	−4.44600	−2.50600
C	−14.10500	−4.75100	−14.60200	−8.35000	−4.66800	−3.78300
C	−15.30000	−5.47500	−15.11500	−7.80300	−3.44800	−4.34300
O	−13.82200	−3.69700	−15.14300	−7.78300	−5.70900	−4.05700
O	−12.64400	−4.56600	−12.77200	−9.54200	−5.40100	−1.90000
C	−12.92800	−3.65000	−11.98500	−9.11200	−5.77500	−0.85900
C	−12.87800	−2.27300	−12.43800	−8.48600	−7.08400	−0.85200
O	−11.93300	−1.76300	−12.87400	−9.17200	−8.10900	−0.97100
C	−12.19600	−3.70600	−10.68400	−9.95100	−5.81900	0.35200
O	−11.01800	−3.60200	−10.75900	−10.92400	−6.51900	0.25100
C	−12.78200	−4.74100	−9.82500	−10.32600	−4.58100	0.87000
C	−12.13700	−6.06500	−9.67600	−11.19200	−3.49700	0.40800
C	−14.16800	−4.62000	−9.32800	−9.38300	−4.07600	1.86300
C	−12.65400	−7.07500	−8.78700	−11.46400	−2.32600	1.17400
C	−14.61400	−5.56100	−8.28200	−9.43300	−2.81200	2.62800
C	−13.99500	−6.87900	−8.30600	−10.28600	−1.84300	1.96300
O	−15.73700	−5.41300	−7.84600	−8.46200	−2.55800	3.28300
C	−15.93700	−4.92400	−6.74900	−8.49300	−2.57900	4.47800
C	−13.04400	−3.54500	−16.07800	−8.13900	−6.26600	−5.04900
H	−15.21800	−8.19900	−13.12300	−9.92800	−1.11400	−2.81700
H	−16.24100	−7.51100	−15.25000	−7.97600	−1.25800	−4.62500
H	−13.69600	−6.71600	−11.71100	−10.76300	−3.19200	−1.70300
H	−15.69800	−5.13200	−16.05600	−7.01900	−3.65700	−5.07900
H	−13.97200	−3.91800	−11.76100	−8.30700	−5.05800	−0.68700
H	−13.22000	−1.56600	−11.70000	−8.01900	−7.12600	0.10700
H	−13.59900	−2.07200	−13.27500	−7.69900	−7.29800	−1.56300
H	−12.40500	−0.97200	−12.59700	−9.46300	−8.15800	−1.81300
H	−12.53400	−2.86100	−10.11800	−9.38300	−6.37700	1.14400
H	−10.62400	−4.36300	−11.17100	−11.28500	−6.41600	−0.63900
H	−11.12300	−6.09500	−10.03400	−11.90400	−3.67300	−0.39400
H	−14.57500	−3.56100	−9.25300	−8.81000	−4.86800	2.38600
H	−12.42100	−8.04800	−9.10300	−12.31700	−1.77000	0.79200
H	−14.35700	−7.67000	−7.62200	−10.43400	−0.87700	2.49600
H	−15.35800	−3.95200	−6.62700	−8.94800	−3.56400	4.78600
H	−16.98000	−4.62800	−6.54600	−7.49000	−2.38900	4.86500
H	−15.53600	−5.55800	−5.97000	−9.10800	−1.90900	4.91500
H	−12.05800	−3.78500	−15.73300	−9.21000	−6.43000	−4.95300
H	−13.33900	−4.08100	−16.98800	−7.94900	−5.60900	−5.86200
H	−13.10100	−2.55800	−16.28200	−7.61900	−7.27100	−5.14200
C	−16.41100	−2.18000	−13.77600	−4.27100	−3.52600	−3.86200
N	−16.19000	−1.53600	−12.59700	−4.68200	−3.38800	−2.58000
C	−16.22300	−2.46400	−11.58800	−4.72100	−2.02000	−2.31300
N	−17.12500	−3.49800	−11.72300	−4.54300	−1.14500	−3.31600
C	−18.01800	−3.39600	−10.68000	−5.47500	−0.06600	−3.15400
N	−17.61700	−2.41300	−9.77600	−6.04600	−0.16600	−1.91800
C	−17.60500	−2.76400	−8.43800	−5.91600	0.93500	−1.01600
C	−16.47200	−1.84800	−10.28200	−5.68600	−1.44500	−1.41100
C	−16.31200	−0.34900	−10.19400	−6.56900	−2.34700	−0.80300
O	−16.68100	0.22600	−9.23500	−7.34700	−1.93400	0.03100
N	−15.63000	0.33100	−11.19900	−6.00400	−3.62700	−0.77100
C	−14.65000	1.28800	−10.88000	−6.28400	−4.44900	0.29300
C	−15.59400	−0.17500	−12.50900	−5.53800	−4.27500	−1.96100
O	−14.82900	0.26500	−13.31700	−5.43900	−5.46100	−2.07900
H	−16.54900	−3.26900	−13.76100	−4.97000	−3.92500	−4.50500
H	−15.69900	−1.98000	−14.55300	−3.53900	−4.33600	−3.89600
H	−17.26200	−1.76800	−14.19300	−3.88300	−2.66700	−4.30200
H	−18.00200	−4.27100	−10.10400	−5.11500	0.94500	−3.33600
H	−18.03600	−3.75300	−8.46900	−6.48100	0.74700	−0.13400
H	−16.59300	−2.59900	−8.19600	−4.96300	1.00100	−0.58000
H	−18.19000	−1.93800	−7.85000	−6.18600	1.79600	−1.60700
H	−13.80500	1.23400	−11.53900	−5.82700	−5.39900	0.27900
H	−14.31500	1.19400	−9.86200	−7.34900	−4.72100	0.33700
H	−15.15400	2.22800	−11.01500	−6.07900	−3.86600	1.17300

**Table A2:** Atomic coordinates of the system consisting of αR, βR guiacyl β−O−4, and caffeine: cluster 3, cluster 4, and cluster 5 structures.

Atomic Coordinates of Gu_Caf Complexes									
	Cluster 3			Cluster 4			Cluster 5		
Atom	x	y	z	x	y	z	x	y	z
C	−13.15800	−6.70000	−15.40500	−8.15800	−1.29400	−15.39700	−10.44800	−5.00000	−6.43400
C	−13.56600	−7.89600	−14.74100	−7.21000	−1.92100	−14.49000	−9.41500	−5.82700	−6.92400
C	−12.79800	−6.74100	−16.82300	−8.30900	−1.84200	−16.71300	−10.23900	−4.51600	−5.11400
C	−13.46400	−7.72000	−17.70300	−8.02500	−3.26300	−16.96100	−8.97200	−4.61300	−4.35000
C	−13.80600	−9.00400	−17.09600	−6.85300	−3.75800	−16.12700	−8.00100	−5.57600	−4.91800
C	−14.13900	−8.94700	−15.61000	−6.80700	−3.32800	−14.73600	−8.07200	−5.86700	−6.31200
O	−14.48100	−9.78500	−17.72000	−6.43800	−4.84400	−16.49100	−6.91500	−5.60100	−4.37100
O	−13.38400	−7.59900	−18.88200	−8.34700	−3.84600	−17.97700	−8.96200	−4.38300	−3.16300
C	−14.26000	−7.00600	−19.49300	−9.06200	−4.84100	−17.92100	−8.37300	−3.42800	−2.74800
C	−14.83000	−7.79200	−20.55600	−8.47400	−6.08800	−18.40000	−7.10900	−3.70900	−2.03800
O	−14.18700	−8.14800	−21.48800	−7.97600	−6.18600	−19.48200	−7.10200	−4.37700	−1.00800
C	−13.91600	−5.68900	−20.04800	−10.38500	−4.81300	−18.59600	−9.12500	−2.51300	−1.88500
O	−13.11800	−5.74200	−20.95500	−10.33300	−4.74500	−19.77700	−9.46300	−3.04600	−0.88600
C	−13.76100	−4.58500	−19.12700	−11.31800	−3.90300	−18.01600	−10.21300	−1.82700	−2.58800
C	−12.44900	−4.16000	−18.79500	−11.45500	−2.56200	−18.67600	−11.54300	−2.47100	−2.84300
C	−14.89500	−4.20400	−18.26700	−11.98000	−4.10700	−16.75100	−9.67900	−0.88800	−3.58700
C	−12.30200	−3.07800	−17.90000	−12.56600	−1.69600	−18.26500	−12.38100	−1.90200	−3.90300
C	−14.78400	−3.06200	−17.32600	−13.12300	−3.19600	−16.34700	−10.53600	−0.39000	−4.63200
C	−13.42500	−2.73000	−17.00100	−13.05600	−1.87800	−16.88700	−11.77100	−1.12400	−4.94900
O	−15.77500	−2.65400	−16.81400	−13.57200	−3.35000	−15.27900	−10.19400	0.45000	−5.41800
C	−16.20700	−1.60700	−17.10100	−14.58700	−3.98300	−15.25600	−10.36500	1.61600	−5.20800
C	−13.98300	−10.74600	−18.24100	−5.34600	−4.94300	−16.91600	−6.44300	−6.46300	−3.71900
H	−12.56400	−5.94200	−14.95700	−8.31000	−0.23500	−15.21900	−11.20100	−4.82900	−7.09500
H	−13.78700	−7.88800	−13.66600	−6.94900	−1.41100	−13.44700	−9.39900	−5.82900	−7.97600
H	−12.61700	−5.79200	−17.39100	−8.91300	−1.30300	−17.37100	−10.97000	−3.72600	−4.78600
H	−14.34200	−9.87600	−15.22600	−6.19700	−3.92300	−14.06900	−7.32000	−6.51400	−6.71700
H	−15.10900	−7.01500	−18.82400	−9.16700	−4.98700	−16.83200	−8.09000	−2.77000	−3.57100
H	−15.72700	−7.35200	−20.97400	−9.13800	−6.92800	−18.24600	−6.39100	−2.92500	−1.85600
H	−15.18800	−8.77800	−20.10800	−7.73700	−6.34500	−17.70700	−6.52600	−4.31900	−2.77800
H	−13.52100	−8.76300	−21.26200	−8.57800	−6.13700	−20.21400	−7.65400	−4.04000	−0.35000
H	−14.88900	−5.31500	−20.46400	−10.73900	−5.74600	−18.36200	−8.42000	−1.72100	−1.60700
H	−13.49200	−6.37700	−21.56900	−11.23300	−4.84900	−20.06400	−10.22500	−3.57200	−1.02600
H	−11.73200	−4.27700	−19.52000	−10.96500	−2.55400	−19.61700	−11.96500	−2.99100	−2.04500
H	−15.87700	−4.39500	−18.58600	−11.93100	−5.06900	−16.25000	−8.78300	−0.42500	−3.42100
H	−11.36100	−2.88100	−17.56500	−12.57600	−0.65600	−18.59300	−13.20000	−2.53300	−4.20500
H	−13.40100	−1.91200	−16.26400	−13.87400	−1.15300	−16.61900	−12.43100	−0.53900	−5.59800
H	−15.40400	−0.93400	−16.86700	−14.53300	−5.02600	−15.56900	−9.92000	1.88300	−4.24400
H	−16.26300	−1.41700	−18.16700	−15.11200	−4.10100	−14.34600	−9.92200	2.22100	−5.90400
H	−17.12100	−1.37800	−16.66800	−15.26100	−3.57400	−15.98600	−11.43500	1.79100	−5.20800
H	−13.19800	−10.49000	−18.97400	−5.32700	−5.86500	−17.45400	−6.94100	−6.64700	−2.83300
H	−13.62500	−11.45700	−17.51100	−5.03500	−4.16400	−17.65200	−6.30400	−7.42900	−4.25100
H	−14.71700	−11.33000	−18.71900	−4.67100	−4.90700	−16.11100	−5.39200	−6.10100	−3.52100
C	−18.09000	−9.45100	−16.01200	−12.45100	−7.26000	−15.14200	−6.67000	1.93500	−5.72000
N	−17.97800	−8.09400	−16.51400	−11.02800	−7.20100	−14.97200	−6.36900	0.56900	−6.13500
C	−17.42700	−7.08100	−15.67400	−10.52400	−6.46400	−13.88100	−5.35500	−0.13600	−5.40400
N	−17.63900	−7.01900	−14.29300	−11.28500	−5.35600	−13.59600	−5.08800	0.01300	−4.04500
C	−18.20100	−5.87400	−13.86900	−10.40100	−4.35200	−13.17500	−5.03900	−1.21800	−3.45300
N	−18.27300	−5.00400	−14.98000	−9.12800	−4.93000	−13.11800	−5.00500	−2.28400	−4.31600
C	−17.88300	−3.71400	−14.72800	−8.62700	−4.79100	−11.80600	−3.91100	−3.12200	−4.19900
C	−17.76500	−5.75200	−16.06500	−9.17000	−6.13700	−13.73700	−5.17900	−1.61600	−5.55600
C	−18.13200	−5.43700	−17.43800	−8.16300	−6.66100	−14.60900	−6.06900	−2.25300	−6.44500
O	−18.37700	−4.34100	−17.86300	−7.02800	−6.63500	−14.24200	−5.85700	−3.44500	−6.64800
N	−18.00600	−6.53000	−18.37700	−8.70300	−7.62000	−15.45200	−7.05200	−1.49800	−7.16800
C	−18.16000	−6.34100	−19.79100	−7.84500	−8.70400	−15.82600	−7.95000	−2.27600	−7.88600
C	−18.02500	−7.86000	−17.90400	−10.04000	−7.75200	−15.80400	−7.29900	−0.14900	−6.91200
O	−17.89000	−8.77700	−18.67500	−10.41500	−8.47000	−16.64800	−8.21000	0.43500	−7.42100
H	−18.30600	−10.16600	−16.85000	−12.88000	−6.61200	−14.34700	−6.65500	1.86000	−4.64200
H	−18.81300	−9.59700	−15.25800	−12.79900	−8.23300	−15.16700	−7.57900	2.23300	−6.29300
H	−17.17700	−9.63200	−15.44700	−12.64300	−6.91900	−16.14500	−5.86800	2.61400	−5.92000
H	−17.53000	−5.45700	−13.14300	−10.68200	−3.92800	−12.17200	−4.11600	−1.19500	−2.85000
H	−17.50600	−3.63500	−13.68500	−7.91800	−3.98400	−11.69600	−3.61700	−3.05700	−3.16300
H	−17.07200	−3.56300	−15.42000	−9.45600	−4.40800	−11.18900	−4.33700	−4.04600	−4.49400
H	−18.63500	−3.10700	−15.08900	−8.18800	−5.65300	−11.27100	−3.11300	−2.91400	−4.85100
H	−18.13300	−7.28000	−20.35300	−7.12300	−8.38900	−16.56000	−8.86200	−2.08900	−7.60300
H	−17.47900	−5.60700	−20.11100	−8.48800	−9.46400	−16.40400	−7.74400	−3.30600	−7.86000
H	−19.05800	−5.82600	−20.05600	−7.44600	−9.26200	−15.00200	−7.84300	−1.93600	−8.95200

**Table A3:** Extended version of Table 3 with calculated energies (counterpoise energy, BSSE, sum of the energy of monomers, counterpoise uncorrected, corrected interaction energies in a vacuum, sum of the energy of monomers and interaction energy in water) for the studied system consisting of αR, βR guiacyl β-O-4, and caffeine.

System Consisted of αR, βR Guiacyl β-O-4 and Caffeine	Vacuum					Water		
	Counterpoise Corrected Energy [Hartree]	BSSE Energy [Hartree]	Sum of Monomers [Hartree]	Interaction Energy (Raw) [kcal/mol]	Interaction Energy (Corrected) [kcal/mol]	Energy [Hartree]	Sum of Monomers [Hartree]	Interaction Energy [kcal/mol]
Clust1	−1715.089733	0.003167	−1715.072141	−13.0	−11.0	−1715.117115	−1715.098914	−11.4
